# Variances of Cylinder Parameters Fitted to Range Data

**DOI:** 10.6028/jres.117.015

**Published:** 2012-09-26

**Authors:** Marek Franaszek

**Affiliations:** National Institute of Standards and Technology, Gaithersburg, MD 20899

**Keywords:** 3D imaging systems, cylinder fitting, directional error function, Nonlinear Least Square fitting, orthogonal error function

## Abstract

Industrial pipelines are frequently scanned with 3D imaging systems (e.g., LADAR) and cylinders are fitted to the collected data. Then, the fitted as-built model is compared with the as-designed model. Meaningful comparison between the two models requires estimates of uncertainties of fitted model parameters. In this paper, the formulas for variances of cylinder parameters fitted with Nonlinear Least Squares to a point cloud acquired from one scanning position are derived. Two different error functions used in minimization are discussed: the orthogonal and the directional function. Derived formulas explain how some uncertainty components are propagated from measured ranges to fitted cylinder parameters.

## 1. Introduction

Digital representation of an object’s surface is important in many engineering applications. Examples include: manufacturing, modular construction, and preservation of historical artifacts. Frequently, the data needed for such representation are collected with 3D imaging systems. The systems are non-contact, line-of-sight instruments which provide range images *r*(*ϑ*, *φ*) of the object’s surface, where *r* denotes the distance (measured as a time of flight) from an instrument to a point on a surface, and *ϑ* and *φ* are the elevation and the azimuth angles to that point. The scanning process is very fast and current systems can collect datasets containing hundreds of thousands of points within a few seconds [1]. Points collected from a surface may be used to build a 3D geometrical model of a scanned object. For example, a cylinder is frequently modeled in projects where industrial pipelines are scanned [2–5]. Frequently, Nonlinear Least Square (NLS) methods [6–10] are used to fit a model to its corresponding point cloud. An important issue is how to propagate an instrument uncertainty (for example: uncertainty of measured ranges *r*) to the uncertainties of fitted cylinder parameters. These uncertainties are essential in a quality control process when the as-built model (derived from the point cloud) is checked against the as-designed (theoretical) model [11]. The tolerances used in the design process and the uncertainties of the fitted model parameters are both needed to accept the as-built model. For example, the uncertainties of the fitted coefficients of the cylinder centerline are needed to check, with a given confidence, if two pipes are parallel or to check the plumbness of a cylinder column.

It is commonly accepted by manufacturers and users of 3D imaging systems that the uncertainty of a *j*-th measured point ***P****_j_*(*X*,*Y*,*Z*) is caused mainly by an uncertainty of the measured range *r_j_*; an impact of angular uncertainty can be neglected and both the elevation and the azimuth angles of ***P****_j_* are treated as noise-free parameters. Under this assumption, the closed formulas for variances (i.e., squared standard deviations) of cylinder parameters fitted to data acquired from one scanning location are provided. These variances are an important component in evaluating measurement uncertainty.

The choice of an error function for NLS minimization is a delicate matter. It was shown that the orthogonal error function, which is commonly used in commercial software, yields results that are in disagreement with experiment when a plane is fitted to a point cloud acquired in some experimental conditions [12]. Another error function, the directional error function, gives results that are, on average, in a much better agreement with experiment. Therefore, in this paper, closed formulas for variances of fitted cylinder parameters are derived for both orthogonal and directional error functions. Both error functions depend on four fitting variables: three angles {*ϑ*, *φ*, *ρ*} which determine a cylinder centerline orientation in space, and the distance *L* of a centerline to the origin of a coordinate system.

## 2. Variances of Fitted Cylinder Parameters

Points ***P****_j_*(*X*, *Y*, *Z*) on a surface of an infinitely long regular cylinder of radius *R* satisfy the following equation

(1) $$\left\|(P_{j}-L)\times w\right\|=R,$$

where the cylinder centerline ***M*** is

(2) M(q)=L(x,y,z)+w(ϑ,φ)q,

***w***(*ϑ*, *φ*) is a unit vector in the direction of the axis, parameterized by two angles: the elevation *ϑ* and the azimuth *φ*, and × stands for a cross product of two vectors. Therefore, the Cartesian coordinates of ***w***(*ϑ*, *φ*) may be written as

(3) w(ϑ,φ)=[cosϑcosφ,cosϑsinφ,sinϑ].

***L*** is any fixed point on the axis and *q* is a number defining a location of arbitrary point on the line ***M***. It is convenient to choose ***L*** in such a way that its length *L* = ‖***L***‖ is the shortest [5]. This happens when ***L*** and ***w*** are perpendicular and their dot product is zero, ***L***·***w*** = 0. In this setting, *L* is the distance of the cylinder centerline ***M*** from the origin of the coordinate system. The origin and the orientation of the coordinate system (in which experimental points ***P****_j_* are acquired) are defined by the location and the orientation of a scanner. Vector ***L*** may be parameterized as

(4a) L=Ll,

where a unit vector ***l*** lies on a plane perpendicular to ***w*** and

(4b) l(ϑ,φ,ρ)=u(ϑ,φ)cosρ+v(ϑ,φ)sinρ.

The unit vectors ***u*** and ***v***, together with the vector ***w***, form the right-handed orthonormal basis {***u***, ***v***, ***w***}

(5a) u=v×w,v=w×u,w=u×v,

(5b) v⋅w=w⋅u=u⋅v=0.

Vectors ***u*** and ***v*** can be parameterized as follows

(6a) v(ϑ,φ)=[sinϑcosφ,sinϑsinφ,−cosϑ],

(6b) u(φ)=[sinφ,−cosφ,0].

**Fig. 1:**
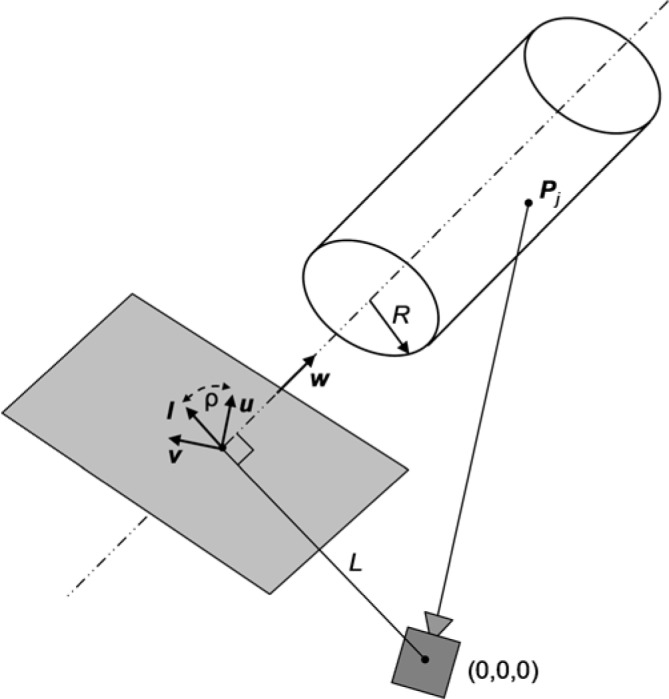
Parameterization of a cylinder for fitting to range data. Unit vectors ***u***,***v***, and ***l*** lie on a plane perpendicular to the unit vector ***w***. Angle *ρ* is measured between vectors ***u*** and ***l***.

The angle *ρ* in Eq. (4b) defines two coordinates of the unit vector ***l*** on the 2D plane perpendicular to ***w***, as shown in Fig. 1. It is easy to check that in this parameterization there are two equivalent sets of parameters defining the same centerline ***M***: {*ϑ*, *φ*, *ρ*, *L*} and {−*ϑ*, *φ* + *π*, *π* − *ρ*, *L*} which correspond to vectors ***w*** and ***−w***.

Point ***P****_j_* from Eq. (1) may be written as

(7) $$P_{j}=r_{j}p_{j}(\vartheta_{j},\varphi_{j}),$$

where *r_j_* is a range and a unit vector ***p****_j_* is equal to

(8) $$p_{j}(\vartheta_{j},\varphi_{j})=[\cos\vartheta_{j}\cos\varphi_{j},\cos\vartheta_{j}\sin\varphi_{j},\sin\vartheta_{j}].$$

It is also convenient to introduce a vector ***d****_j_*

(9) $$d_{j}(\vartheta ,\varphi ,\vartheta_{j},\varphi_{j})=p_{j}(\vartheta_{j},\varphi_{j})\times w(\vartheta ,\varphi ).$$

Now, using the above introduced vector ***d****_j_* together with Eqs. (4) and (5), Eq. (1) can be expressed as

(10) $$\left\|r_{j}d_{j}-L(u\sin\rho -v\cos\rho )\right\|=R.$$

**Fig. 2:**
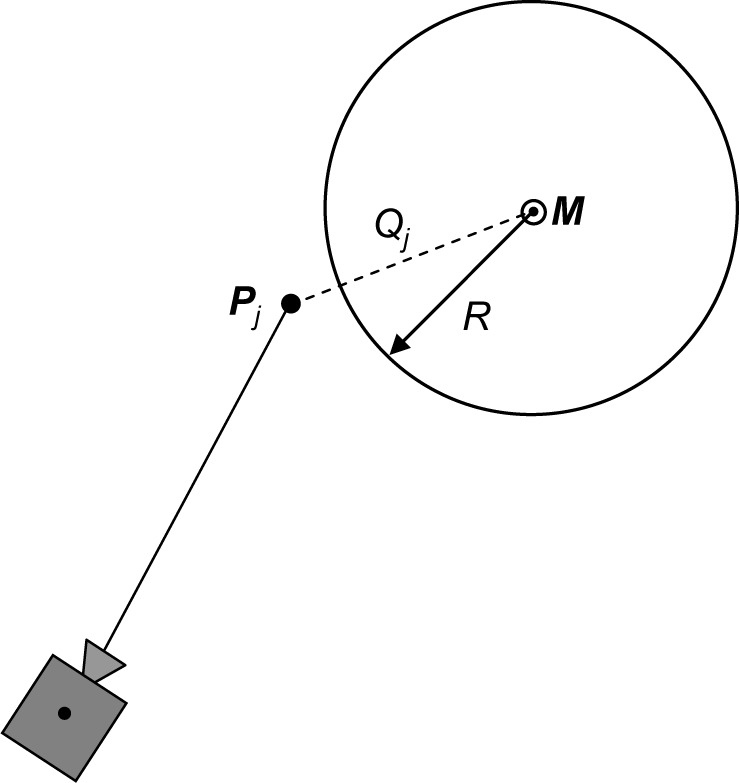
Schematic drawing (top view, along the cylinder centerline ***M***) explaining the calculation of the orthogonal error function.

**Fig. 3:**
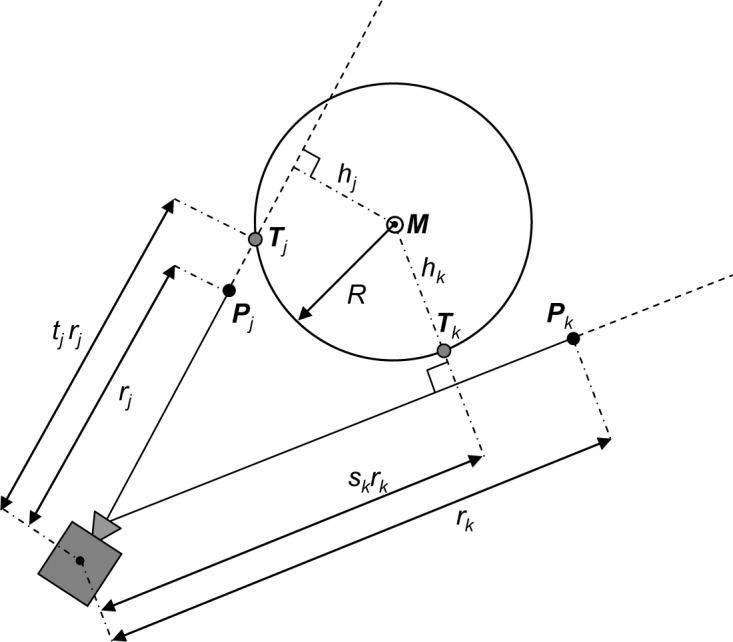
Schematic drawing (top view, along the cylinder centerline ***M***) explaining the calculations of the directional error function. Point ***P****_j_* illustrates the configuration when a ray intersects with the cylinder surface (*h_j_* < *R*) while point ***P****_k_* illustrates when there is no intersection (*h_k_* > *R*).

Thus, in this parameterization, a regular cylinder of radius *R* is defined by four parameters: three angles *ϑ*, *φ*, *ρ* and the distance *L* (in this and the next two sections, a radius *R* is treated as a known constant; later in Section V this restriction is removed for the orthogonal fitting). Due to imperfections in range measurement, experimental points ***P****_j_* do not lie exactly on a cylinder surface. Therefore, a model of a cylinder has to be fitted to the experimental dataset ***P****_{N}_* = {***P****_j_*, *j* = 1,…, *N*}, where *N* denotes the number of points. Within the framework of the Least Squares method, the estimated parameters of a cylinder {ϑ^*^, φ^*^, ρ^*^, L^*^} are obtained by minimizing the error function

(11) $$E(\vartheta ,\varphi ,\rho ,L,P_{\left\{N\right\}})=\frac{1}{N}\sum_{j=1}^{N}E_{j}(\vartheta ,\varphi ,\rho ,L,P_{j}),$$

where *E_j_* is the squared distance between the experimental point ***P****_j_* and its corresponding theoretical point on a cylinder surface. Different definitions of the theoretical point yield different error functions. In this paper we study two error functions: the orthogonal error function *E_O_* and the directional error function *E_D_*, as explained in Figs. 2 and 3 and in the next two sections. The elements of a gradient of the error function ∇*E* can be calculated as

(12) $$\frac{\partial E}{\partial \alpha }(\vartheta ,\varphi ,\rho ,L,P_{\left\{N\right\}})=\frac{1}{N}\sum_{j=1}^{N}\frac{\partial E_{j}}{\partial \alpha }(\vartheta ,\varphi ,\rho ,L,P_{j}),\;\text{for}\;\alpha =(\vartheta ,\varphi ,\rho ,L).$$

The knowledge of closed formulas for a gradient ∇*E* allows application of efficient minimization algorithms to find the fitted parameters {ϑ^*^, φ^*^, ρ^*^, L^*^}. Their values depend solely on the experimental dataset ***P****_{N}_*, i.e.,

(13) $$\vartheta^{*}=\vartheta^{*}(P_{\left\{N\right\}}),\;\varphi^{*}=\varphi^{*}(P_{\left\{N\right\}}),\;\rho^{*}=\rho^{*}(P_{\left\{N\right\}}),\;L^{*}=L^{*}(P_{\left\{N\right\}}).$$

The variances of the fitted cylinder parameters var(ϑ^*^), var(φ^*^), var(ρ^*^), and var(L^*^) may be calculated following the same general approach developed originally for fitting a sphere to range data [13]. Here, similarly as in the previous study, only range uncertainty is considered and the bearings (*ϑ_j_*, *φ_j_*) of acquired *j*-th point ***P****_j_* are treated as noise-free parameters. For other types of instruments, for example coordinate measuring machines (CMM), the above assumption may not be valid and the formulas for variances of fitted cylinder parameters developed in this paper may not be applicable. In addition, it is assumed that measured range *r_j_* is not correlated with *r_k_* for any *j* ≠ *k*. When both assumptions are valid, the variances of the fitted cylinder parameters may be estimated by applying to Eq. (13) the uncertainty propagation formula [14]

(14) $$\mathrm{var}(\alpha^{*})={\sum_{j=1}^{N}\left[\frac{\partial \alpha^{*}\left(P_{\left\{N\right\}}\right)}{\partial r_{j}}\right]}^{2}\mathrm{var}(r_{j}),\;\text{for}\;\alpha^{*}=\{\vartheta^{*},\varphi^{*},\rho^{*},L^{*}\}.$$

For every *j*, the sensitivity vector ***S****_j_*(***P***_{_*_N_*_}_)

(15) $$S_{j}(P_{\left\{N\right\}})=\left[\begin{array}{c} \frac{\partial \vartheta^{*}}{\partial r_{j}}(P_{\left\{N\right\}})\\ \frac{\partial \varphi^{*}}{\partial r_{j}}(P_{\left\{N\right\}})\\ \frac{\partial \rho^{*}}{\partial r_{j}}(P_{\left\{N\right\}})\\ \frac{\partial L^{*}}{\partial r_{j}}(P_{\left\{N\right\}}) \end{array}\right]$$

can be evaluated by solving the following 4×4 system of linear equations

(16) $$H(\vartheta^{*},\varphi^{*},\rho^{*},L^{*},P_{\left\{N\right\}})S_{j}(P_{\left\{N\right\}})=-V_{j}(\vartheta^{*},\varphi^{*},\rho^{*},L^{*},P_{j})$$

where the vector ***V****_j_* is defined by

(17) $$V_{j}(\vartheta ,\varphi ,\rho ,L,P_{j})=\frac{\partial }{\partial r_{j}}\nabla E(\vartheta ,\varphi ,\rho ,L,P_{\left\{N\right\}})$$

while ***H*** is 4×4 Hessian matrix which elements are given by

(18) $$H_{\alpha ,\beta }(\vartheta ,\varphi ,\rho ,L,P_{\left\{N\right\}})=\frac{\partial^{2}E}{\partial \alpha \;\partial \beta }(\vartheta ,\varphi ,\rho ,L,P_{\left\{N\right\}})\;\text{for}\;\alpha ,\beta =\{\vartheta ,\varphi ,\rho ,L\}.$$

## 3. Orthogonal Fitting

When fitting a cylinder using the orthogonal error function (see Fig. 2) the theoretical point corresponding to the experimental point ***P****_j_* is defined as the orthogonal projection of ***P****_j_* on a cylinder surface. Thus, Eq. (11) takes the form

(19) $$E_{O}(\vartheta ,\varphi ,\rho ,L,P_{\left\{N\right\}})=\frac{1}{N}\sum_{j=1}^{N}{\left(Q_{j}-R\right)}^{2}$$

where *Q_j_* is a distance of point ***P****_j_* to the cylinder axis ***M***, which can be evaluated from Eq. (10)

(20) $$Q_{j}(\vartheta ,\varphi ,\rho ,L,P_{j})=\sqrt{r_{j}d_{j}-L(u\sin\rho -v\cos\rho )]\cdot [r_{j}d_{j}-L(u\sin\rho -v\cos\rho )]}.$$

Defining the scalar functions *a_j_* and *b_j_* by means of Eqs. (3), (5), (8), (9) and using the properties of the dot and cross products,

(21a) $$a_{j}(\vartheta ,\varphi ,p_{j})=d_{j}\cdot d_{j}=1-{\left(w\cdot p_{j}\right)}^{2},$$

(21b) $$b_{j}(\vartheta ,\varphi ,\rho ,p_{j})=p_{j}\cdot l=p_{j}\cdot \left(v\sin\rho +u\cos\rho \right),$$

the distance *Q_j_* from Eq. (20) may be written as

(22) $$Q_{j}(\vartheta ,\varphi ,\rho ,L,P_{j})=\sqrt{a_{j}(\vartheta ,\varphi ,p_{j})r_{j}^{2}-2Lr_{j}b_{j}(\vartheta ,\varphi ,\rho ,p_{j})+L^{2}}.$$

Explicit expressions for the gradient ∇*E_O_* may be obtained by inserting Eq. (22) into Eq. (19) and using Eq. (12). The minimum $E_{O}(\vartheta_{O}^{*},\varphi_{O}^{*},\rho_{O}^{*},L_{O}^{*})$ can be found numerically using gradient ∇*E_O_*. Then, Eqs. (17) and (18) can be applied to Eq. (19) to get the vector ***V****_j_* and the matrix ***H****_O_*. Once the sensitivity vector ***S****_j_* is evaluated from Eq. (16), the variances of fitted cylinder parameters $\mathrm{var}\left(\alpha_{O}^{*}\right)$ for $\alpha_{O}^{*}=\{\vartheta_{O}^{*},\varphi_{O}^{*},\rho_{O}^{*},L_{O}^{*}\}$ can be calculated from Eq. (14).

## 4. Directional Fitting

When fitting a cylinder using the directional error function, see Fig. 3, the theoretical point ***T****_j_* corresponding to the experimental point ***P****_j_* is defined as an intersection of a ray originating from the instrument and passing through ***P****_j_* with the cylinder surface

(23) $$T_{j}=t_{j}P_{j},$$

where the function *t_j_*(*ϑ*, *φ*, *ρ*, *L*, ***P****_j_*) has its value close to one when optimization variables are close to their best-fit values and the theoretical points ***T****_j_* satisfy Eq. (1) of the cylinder

(24) $$\left\|(T_{j}-L)\times w\right\|=R.$$

A ray need not always intersect a cylinder surface – a similar situation occurs in the directional fitting of a sphere [15]. Whether there is an actual intersection or not can be checked by calculating the distance *h_j_* between the cylinder centerline ***M*** given by Eq. (2) and a ray passing through ***P****_j_*; if *h_j_* < *R*, then there is an intersection and the theoretical point on a cylinder surface is defined by Eq. (23). Otherwise, if *h_j_* ≥ *R*, there is no intersection and the theoretical point ***T****_j_* is defined as a point on a cylinder surface which has the smallest distance to a ray passing through ***P****_j_*. Assuming that a ray is not parallel to the cylinder centerline ***M*** (in which case *h_j_* = *L*), the distance function *h_j_*(*ϑ*, *φ*, *ρ*, *L*, ***p****_j_*) can be calculated as

(25) $$h_{j}(\vartheta ,\varphi ,\rho ,L,p_{j})=\left|L\cdot (p_{j}\times w)\right|/\left\|p_{j}\times w\right\|.$$

Using a definition of the vectors ***L***, ***l***, ***w***, ***v*** and ***u*** given in Eqs. (3)–(6), function *a_j_* in Eq. (21a) and the property of the triple vector product, the distance function *h_j_* can be evaluated as

(26) $$h_{j}(\vartheta ,\varphi ,\rho ,L,p_{j})=L\left|p_{j}\cdot (v\cos\rho -u\sin\rho )\right|/\sqrt{a_{j}}.$$

The *j*-th term in Eq. (11) corresponding to the directional error function *E_D_* is a squared distance between the experimental point ***P****_j_* and the theoretical point ***T****_j_*, see Fig. 3, giving

(27) $$E_{j}(\vartheta ,\varphi ,\rho ,L,P_{j})=(T_{j}-P_{j})\cdot (T_{j}-P_{j}).$$

For the case when *h_j_* < *R*, *E_j_* can be evaluated using the function *t_j_* (*ϑ*, *φ*, *ρ*, *L*, ***P****_j_*) which is derived in the Appendix, Eqs. (A1)–(A5). When *h_j_* ≥ *R*, *E_j_* can be evaluated using *h_j_*(*ϑ*, *φ*, *ρ*, *L*, ***p****_j_*) from Eq. (26) and *s_j_*(*ϑ*, *φ*, *ρ*, *L*, ***P****_j_*) which is derived in the Appendix, Eqs. (A6)–(A10), giving

(28) $$E_{j}(\vartheta ,\varphi ,\rho ,L,P_{j})=\{\begin{array}{cc} {\left(t_{j}-1\right)}^{2}r_{j}^{2} & if\;h_{j}<R\\ {\left(h_{j}-R\right)}^{2}+{\left(s_{j}-1\right)}^{2}r_{j}^{2} & if\;h_{j}\geq R \end{array}.$$

From Eq. (28), the *j*-th term in Eq. (12) for the components of the gradient of the directional error function ∇*E_D_* can be evaluated

(29) $$\frac{\partial E_{j}}{\partial \alpha }=\{\begin{array}{cc} 2r_{j}^{2}(t_{j}-1)\frac{\partial \;t_{j}}{\partial \alpha } & if\;h_{j}<R\\ 2r_{j}^{2}(s_{j}-1)\frac{\partial s_{j}}{\partial \alpha }+2(h_{j}-R)\frac{\partial \;h_{j}}{\partial \alpha } & if\;h_{j}\geq R \end{array}\;\text{for}\;\alpha =\{\vartheta ,\varphi ,\rho ,L\}.$$

The minimum $E_{D}(\vartheta_{D}^{*},\varphi_{D}^{*},\rho_{D}^{*},L_{D}^{*})$ can be found numerically using gradient ∇*E_D_*. Then, Eqs. (17) and (18) can be applied to *E_D_* and Eq. (29) to get the equations for the vector ***V****_j_* and the Hessian matrix ***H****_D_*. Once the sensitivity vector ***S****_j_* is evaluated from Eq. (16), the variances of fitted cylinder parameters $\mathrm{var}\left(\alpha_{D}^{*}\right)$ for $\alpha_{D}^{*}=\{\vartheta_{D}^{*},\varphi_{D}^{*},\rho_{D}^{*},L_{D}^{*}\}$ can be calculated from Eq. (14).

The derivatives of *t_j_*, *s_j_*, and *h_j_* can be calculated from their definitions. Although the derivative of *h_j_* is not defined when *h_j_* = 0, *h_j_* enters into a *j*-th term of *E_D_* only when *h_j_* > *R*, as follows from Eq. (28). Thus, *h_j_* is differentiable in the whole domain where experimental conditions are valid.

## 5. Discussion

So far, only four fitting variables {*ϑ*, *φ*, *ρ*, *L*} were used and the radius of a cylinder *R* was treated as a known constant. When the radius is unknown and needs to be fitted, the orthogonal error function *E_O_* in Eq. (19) may be still effectively minimized in four dimensional space {*ϑ*, *φ*, *ρ*, *L*} [5]. This is possible because the radius *R* enters Eq. (19) in a quadratic term. Since a gradient of the error function must be zero when the minimum of *E_O_* is reached, the radius *R* can be evaluated from the condition

(30) $$\frac{\partial E_{O}}{\partial R}\left(\vartheta ,\varphi ,\rho ,L,R,P_{\left\{N\right\}}\right)=0.$$

Substituting for *E_O_* Eq. (19), the radius *R* may be calculated as

(31) $$R\equiv R(\vartheta ,\varphi ,\rho ,L,P_{\left\{N\right\}})=\frac{1}{N}\sum_{j=1}^{N}Q_{j}\left(\vartheta ,\varphi ,\rho ,L,P_{j}\right),$$

where *Q_j_* is calculated in Eq. (22). Equation (31) has a simple geometric interpretation: the fitted radius R^*^ is the algebraic mean of distances of all experimental points ***P****_j_* from the fitted cylinder centerline defined by $\left\{\vartheta^{*},\varphi^{*},\rho^{*},L^{*}\right\}$. The variance of the fitted radius $\mathrm{var}\left(R^{*}\right)$ can be calculated by applying to Eq. (31) the uncertainty propagation formula [14]

(32) $$\mathrm{var}(R^{*})=\sum_{\alpha }{\left(\frac{\partial R}{\partial \alpha }\right)}^{2}\mathrm{var}(\alpha^{*})+\sum_{j=1}^{N}{\left(\frac{\partial R}{\partial r_{j}}\right)}^{2}\mathrm{var}(r_{j})\;\text{for}\;\alpha =\{\vartheta ,\varphi ,\rho ,L\}.$$

Individual derivatives $\frac{\partial R}{\partial \alpha }$ are calculated at fitted values $\alpha^{*}=\{\vartheta^{*},\varphi^{*},\rho^{*},L^{*}\}$ by inserting Eq. (31) into (32) and using derivatives of *Q_j_*. Individual variances var (α^*^) of the fitted parameters are calculated in Eq. (14) using the formulas derived in Section III for the orthogonal fitting.

Unfortunately, the above strategy for fitting the unknown cylinder radius *R* cannot be applied to the directional fitting because a *j*-th term in the directional error function *E_D_* depends on *R* in a more complex way and a convenient separation of fitting variables as in Eq. (31) is not possible.

Closed formulas for variances derived in this paper are applicable to a cylinder fitted to a point cloud acquired from only one scanning location. When two or more datasets acquired from different locations are registered together, the formulas presented here cannot be used. This limitation has its root in the way range uncertainties are propagated into uncertainties of fitted parameters: the registration process makes relatively straightforward formulas much more complicated, with variances explicitly depending on the registration parameters. It remains an open question whether fitting a model to one large point cloud consisting of many registered datasets has an advantage over fitting a model to many unregistered clouds.

The difference between the orthogonal and the directional fitting becomes more important for point clouds containing many points acquired at large angle of incidence (i.e., AOI - the angle between a ray coming through the data point ***P****_j_* and a normal to the cylinder surface at the theoretical point ***T****_j_* defined in Eq. (23)). Scanning industrial pipelines (where a length of a scanned pipe may be larger than the fitting variable *L*) may yield a dataset in which AOIs are varying in a large range. Then, the orthogonal fitting may generate a cylinder centerline which is biased and incorrect. In order to quantify the expected differences between both error functions, further research involving computer simulations and field measurements is needed.

## 6. Appendix

This Appendix contains expressions needed for evaluating the directional error function *E_D_*, defined in Eq. (28). The individual *j*-th term of *E_D_* depends on the distance *h_j_* between the cylinder centerline ***M*** and a ray passing through data point ***P****_j_*. Let us first consider the case when *h_j_*, given by Eq. (26), is less than the cylinder radius *R*. Eqs. (27) and (24) lead to the following equation for *t_j_*

(A1) $$\left\|(t_{j}P_{j}-L)\times w\right\|=\sqrt{\left[(t_{j}P_{j}-L)\times w]\cdot [(t_{j}P_{j}-L)\times w\right]}=R,$$

where *t_j_* depends on ***P****_j_* and parameters determining ***M***, i.e., *ϑ*, *φ*, *ρ*, and *L*. By squaring both sides of Eq. (A1) and using the definitions of *a_j_* and *b_j_* from Eqs. (21a) and (21b), the following quadratic equation for *t_j_* is obtained

(A2) $$(a_{j}r_{j}^{2})t_{j}^{2}-(2r_{j}Lb_{j})t_{j}+L^{2}-R^{2}=0.$$

This quadratic equation has two solutions. From a definition of the theoretical point ***T****_j_* in Eq. (23), it follows that only *t_j_* > 0 has a physical meaning. Two different experimental settings for collecting data points ***P****_{N}_* need to be distinguished: a) when a cylinder (for example, a pipe) is scanned from the outside; and b) when scanning is done from inside (for example, in a tunnel or inside a containment vessel for a nuclear reactor).

In the first case, *L* > *R* and *b_j_* defined in Eq. (21b) is always positive (*a_j_* from Eq. (21a) is always positive, regardless of a scanner location). Then, from the two real solutions of Eq. (A2), a physical meaning exists only for the smaller one which corresponds to the intersection of a ray with a cylinder surface closer to a scanner location (the origin of coordinates), as shown in Fig. 3. It is useful to define an auxiliary function *Δ_j_*(*ϑ*, *φ*, *ρ*, *L*, ***p****_j_*) as

(A3) $$\Delta_{j}(\vartheta ,\varphi ,\rho ,L,p_{j})=L^{2}b_{j}^{2}-a_{j}(L^{2}-R^{2}).$$

Substituting *Δ_j_* in the solution of quadratic Eq. (A2) yields the expression for *t_j_* as a function of experimental point ***P****_j_* and fitting variables

(A4) $$t_{j}(\vartheta ,\varphi ,\rho ,L,P_{j})=\frac{1}{a_{j}r_{j}}(Lb_{j}-\sqrt{\Delta_{j}}).$$

In the second case, when scanning is done from inside a cylinder and *L* < *R*, *Δ_j_* in Eq. (A3) is always positive and $\sqrt{\Delta_{j}}>Lb_{j}$. However, the sign of *b_j_* may now vary. Since the requirement that *t_j_* is positive must be uphold, the solution for *t_j_* takes now the following form

(A5) $$t_{j}(\vartheta ,\varphi ,L,P_{j})=\frac{1}{a_{j}r_{j}}(Lb_{j}+\sqrt{\Delta_{j}}).$$

Once *t_j_* is known, a *j*-th term in the directional error function *E_D_* can be calculated for the case when *h_j_* < *R* according to Eq. (28).

When *h_j_* ≥ *R*, scanning can be done only from outside and a ray passing through ***P****_j_* does not intersect with a cylinder surface; then quadratic Eq. (A2) does not have a real solution and *t_j_* cannot be determined. In such a case, a point ***G****_j_* on a ray is sought which has the smallest distance to the cylinder centerline ***M***, that is

(A6) $$G_{j}=s_{j}P_{j}.$$

The actual value of *s_j_* can be evaluated by finding a minimum of a squared distance of ***G****_j_* to ***M***,

(A7) $$U_{j}(s_{j})={\left\|(s_{j}P_{j}-L)\times w\right\|}^{2}=\left[(s_{j}P_{j}-L)\times w\right]\cdot [(s_{j}P_{j}-L)\times w],$$

where ***w*** is given by Eq. (3) and ***L*** by Eqs. (4a) and (4b). Using the properties of multiple dot and cross products, Eqs. (5), (6), and (21), *U_j_* can be expressed as

(A8) $$U_{j}(s_{j})=a_{j}s_{j}^{2}r_{j}^{2}+L^{2}-2s_{j}r_{j}Lb_{j}.$$

For fixed cylinder parameters *ϑ*, *φ*, *ρ*, *L* and a given data point ***P****_j_*, a minimum of *U_j_* can be found from the condition

(A9) $$\frac{\partial \;U_{j}}{\partial \;s_{j}}=0.$$

Substituting Eq. (A8) into (A9), the explicit dependence of *s_j_* on fitting variables can be determined

(A10) $$s_{j}(\vartheta ,\varphi ,\rho ,L,P_{j})=L\frac{b_{j}}{r_{j}a_{j}}.$$

The distance *h_j_* defined in Eq. (26) together with *s_j_* provided in Eq. (A10) are used to calculate a *j*-th term in the directional error function *E_D_* when *h_j_* ≥ *R*, according to Eq. (28).
